# IgLON5-Associated Encephalitis With Atypical Brain Magnetic Resonance Imaging and Cerebrospinal Fluid Changes

**DOI:** 10.3389/fneur.2018.00329

**Published:** 2018-05-17

**Authors:** Massimiliano Montagna, Rizvana Amir, Ilse De Volder, Martin Lammens, Jef Huyskens, Barbara Willekens

**Affiliations:** ^1^Department of Neurology, Antwerp University Hospital, Antwerp, Belgium; ^2^Master After Master in Medicine, Faculty of Medicine and Health Sciences, University of Antwerp, Antwerp, Belgium; ^3^Department of Neurology, Sint Maarten General Hospital, Mechelen, Belgium; ^4^Multidisciplinary Sleep Disorders Centre, Antwerp University Hospital, Antwerp, Belgium; ^5^Department of Pathology, Antwerp University Hospital, University of Antwerp, Antwerp, Belgium; ^6^Department of Radiology, Antwerp University Hospital, Antwerp, Belgium; ^7^Laboratory of Experimental Hematology, Faculty of Medicine and Health Sciences, University of Antwerp, Antwerp, Belgium

**Keywords:** brain inflammation, IgLON5, autoimmune encephalitis, rapidly evolving dementia, akathisia, dyskinesia

## Abstract

IgLON5-associated encephalitis is a syndrome with different clinical presentations consisting of sleep dysfunction, bulbar dysfunction, chorea, and progressive supranuclear palsy-like symptoms whereas dysautonomy and cognitive decline usually appear in later stages of the disease. We report a case of a patient with IgLON5-associated encephalitis presenting with rapidly progressive cognitive decline and atypical inflammatory lesions on brain magnetic resonance imaging, oligoclonal bands on cerebrospinal fluid, anti-IgLON5 antibodies exclusively of the IgG1 class, and a fierce inflammatory reaction on brain biopsy, who responded favorably to immunotherapy.

## Introduction

**Figure 1:**
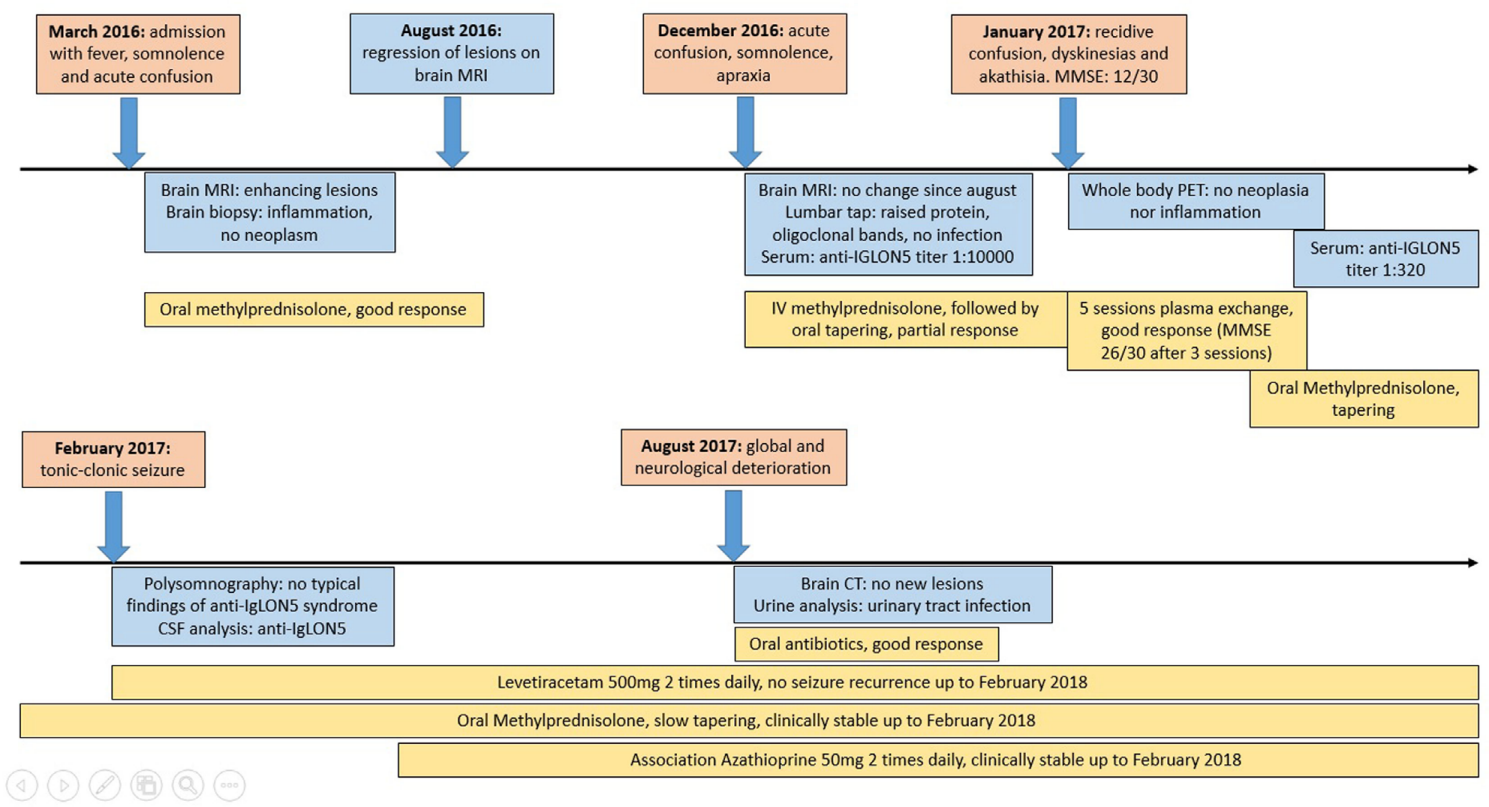
A timeline displaying synthetically the evolution of our case: in the red boxes, the clinical events have been reported; in the blue boxes, there is a list of the significant investigations that have been performed; and in the yellow boxes, we displayed the therapies and the relative effect.

**Figure 2:**
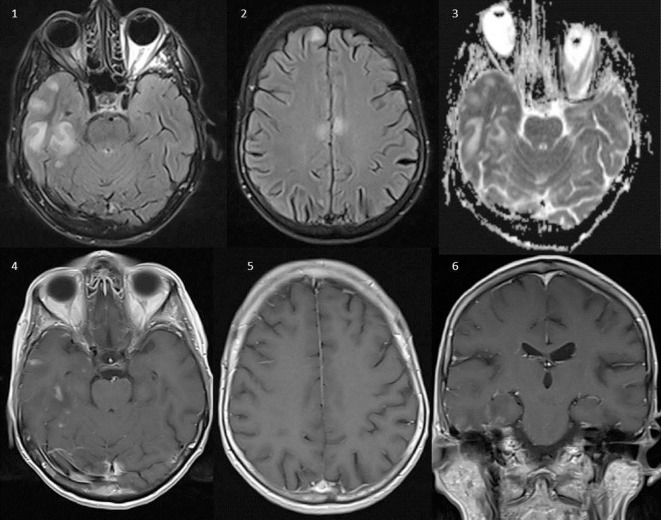
Brain magnetic resonance imaging (Siemens Aera 1.5 T). Axial FLAIR (1, 2), axial ADC-maps (3), axial T1 after intravenous gadolinium (4, 5), and coronal T1 after intravenous gadolinium (6). Several lesions with high T2-signal on FLAIR in the right temporal lobe, bilateral in the frontal lobe, and the callosal body without signs of restricted diffusion on the ADC-maps, compatible with vasogenic edema. Several lesions in the right temporal lobe show patchy contrast enhancement after intravenous administration of gadolinium-based contrast (4–6). None of the lesions were hemorrhagic.

**Figure 3:**
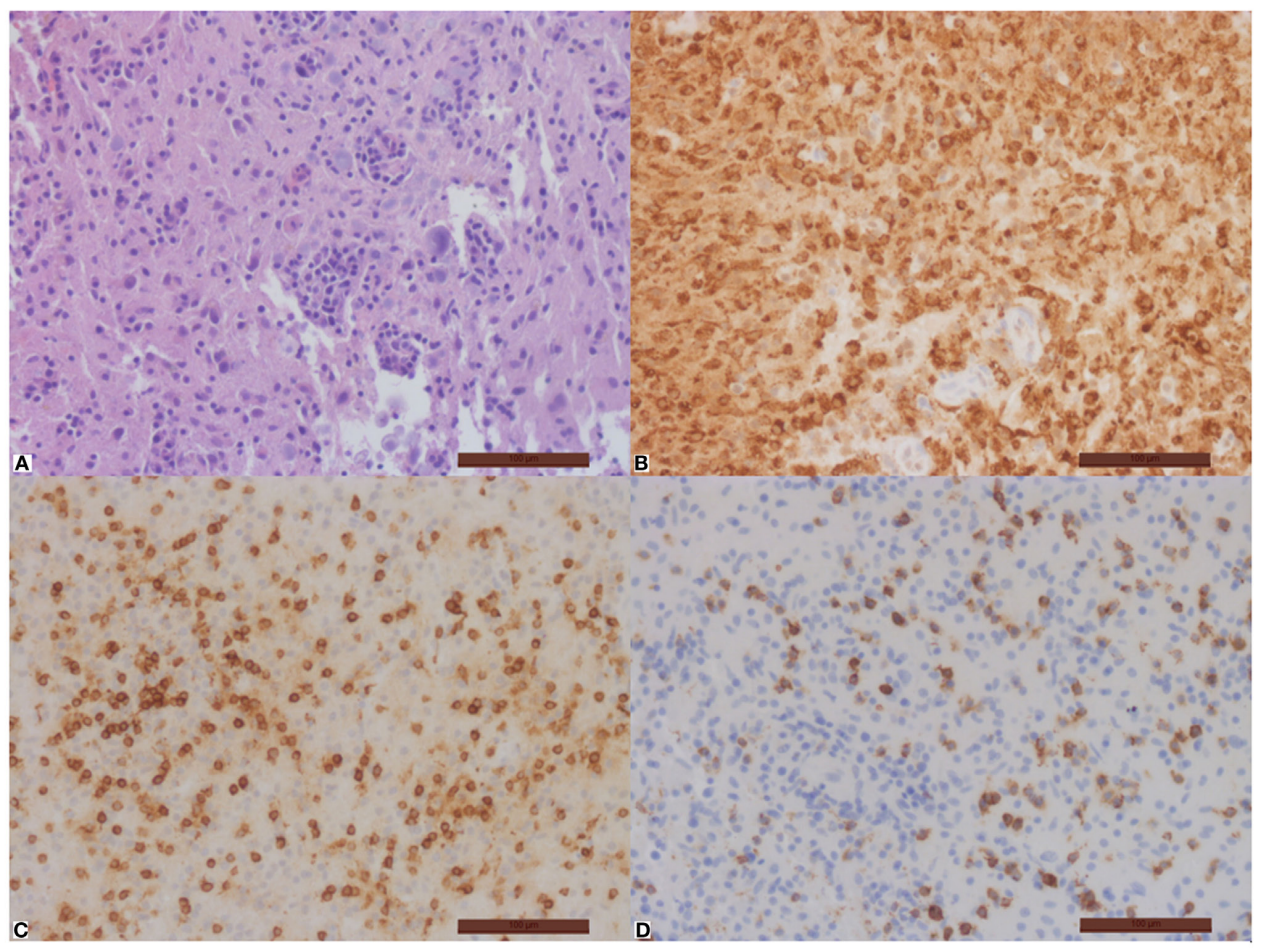
Important inflammation of the brain tissue with disseminated macrophages **(B)** and T-cells **(C)**, no B-cells. Part of the T-cells is CD8-positive **(D)**. Hematoxylin–eosin **(A)**, immunohistochemical stain with antibodies against CD68 **(B)**, CD3 **(C)**, and CD8 **(D)**. Magnification bar = 100 µm.

A 75-year-old female patient was admitted to the geriatric ward in March 2016 with acute confusion, somnolence, verbal aggression, and fever (see Figure 1 for a timeline of this case report). Brain magnetic resonance imaging (MRI) showed spotty enhancement in the right temporal and frontal lobes with focal leptomeningeal enhancement and edema (Figure 2). An extensive workup under suspicion of leptomeningeal metastasis was negative for a primary neoplasm. A brain biopsy performed to exclude a lymphoma showed signs of severe white matter destruction with many macrophages and lymphocytosis, but no malignancy, nor signs of vasculitis (Figure 3). Neurological consultation nor cerebrospinal fluid (CSF) analysis was performed at this stage. Oral corticosteroid treatment led to significant regression of the symptoms, and a follow-up MRI in August 2016 showed a significant decrease in the volume of the lesions with disappearance of gadolinium enhancement.

During a second episode of acute confusion, apraxia, visual hallucinations, and somnolence in December 2016, brain MRI remained unchanged in comparison to the brain MRI performed in August. Electro-encephalography (EEG) showed large amounts of delta waves but no epileptic activity. CSF analysis showed a normal cell count, a mildly elevated protein level (48.4 mg/dl) and 15 oligoclonal bands of which 3 were matched between serum and CSF. Polymerase chain reaction analysis on CSF showed no evidence for the presence of DNA of herpes simplex 1 and 2 virus and varicella zoster virus. Metabolic and infectious etiologies were ruled out. Under suspicion of an autoimmune encephalitis, anti-IgLON5 antibodies were detected in the serum (titer 1:10,000) while other autoantibodies remained negative [the antibodies against the following antigens were tested: Hu, Yo, Ri, CV2, amphiphysin, Ma2/Ta, Zic4, GAD65, Tr(DNER), Recoverin, Sox1—method: EUROLINE; MOG, NMDA-r, AMPA-r, GABA-b, LGI-1, CASPR2, DPPX, myelin, glycine receptors, mGluR1, mGluR5, GABA-a, Rho GTAase activating protein 26, CARPVIII, GluRD2, flotillin—method: immunofluorescence test] (Euroimmun AG, Lübeck, Germany).

High-dose intravenous methylprednisolone (1 g daily for 3 days) leads to a moderate improvement of the consciousness level and apraxia but had only minor effects on the hallucinations. Oral steroids were tapered slowly over several weeks, but in January 2017 she relapsed. On examination, she was logorrheic, incoherent with lower limb dyskinesias and akathisia. The Mini Mental State Examination (MMSE) score was 12/30. A whole body positron emission tomography/computed tomography scan showed no evidence of inflammation or neoplasia.

Five sessions of plasma exchange were performed: after three sessions the MMSE improved to a score of 26/30, hallucinations and dyskinesias completely disappeared. The titer of anti-IgLON5 antibodies decreased to 1:320 after this treatment. One month after discharge the patient was readmitted due to a tonic–clonic seizure for which levetiracetam was started. A polysomnographic examination (PSG) was performed but showed no evidence for stridor, finalistic movements, or repetitive rapid periodic leg movements.

Cerebrospinal fluid analysis, performed at this point, showed the presence of anti-IgLON5 antibodies. The presence of the anti-IgLON5 antibodies on serum and CSF was confirmed in the laboratory of Prof. Dr. Dalmau and Prof. Dr. Graus [Institut d’Investigacions Biomèdiques August Pi i Sunyer (IDIBAPS), Barcelona]. No other autoantibodies were detected. Using previously reported techniques (1), IgLON5 antibodies of the patient recognized an epitope in the Ig-like domain 2 of IgLON5. The IgG antibody subclass was exclusively IgG1. A re-examination of the brain biopsy showed no presence of Tau-protein deposition. HLA typing showed that our patient had haplotype DQB1*0501 and DRB1*1001.

After a new corticosteroid tapering regimen and maintenance treatment with azathioprine 50 mg two times daily our patient has remained neurologically stable with no more hallucinations nor movement disorders and a slight cognitive impairment. A brain CT was performed in June 2017 due to transient neurological regress (later proved to be caused by a urinary tract infection and completely resolved after appropriate antibiotic treatment): this investigation showed no new pathological findings. After this date, no more brain imaging study was performed.

She was still ambulatory up to November 2017 but due to social reasons and general frailty with increasing help demand she was finally admitted to a nursing home. A follow-up contact in February 2018 showed a still remarkable cognitive function (MMSE score 27/30) and no evidence for relapse of epilepsy. Our patient had still episodes of visual hallucinations in the period before being admitted to the nursing home: there were then issues of probable suboptimal therapeutic compliance. After being admitted, compliance improved and visual hallucinations disappeared. At the moment of the last contact, our patient was on methylprednisolone 4 mg daily and azathioprine 50 mg two times per day orally.

## Discussion

Anti-IgLON5 syndrome was first described in 2014 (2) as a disorder characterized by sleep dysfunction, a progressive supranuclear palsy-like syndrome (3), movement disorders (e.g., chorea) and brainstem and hypothalamic involvement leading to dysphagia and dysarthria, with a varying degree of dysautonomic features. Cognitive decline has been described mostly in a later stage of the disease. Neuropathologic findings show tau deposits in the hypothalamus and tegmentum: the relation with anti-IgLON5 has been suspected but not clarified. HLA-DRB1*1001 and HLA-DQB1*0501 association suggests an autoimmune pathogenesis (4).

In all IgLON5-positive patients PSG shows various anomalies such as abnormal sleep architecture, undifferentiated non rapid eye movement (non-REM) sleep or poorly structured stage N2, REM sleep behavior disorder, central hypoventilation, stridor, and obstructive sleep apnea. No significant abnormalities have been found on EEG, electromyography (2) and brain MRI, with the exception of slight brainstem and bilateral hippocampal atrophy (described, respectively, in three patients and one patient) (3). No clear association with an underlying neoplastic pathology has been found thus far. CSF analysis varies from normal (2), to pleiocytosis and increased protein levels (5). Only in one previously reported patient intrathecal synthesis of immunoglobulins has been described (3). Treatment with immunosuppressants showed highly variable results, with a tendency for improved response after earlier start of the treatment (2, 6, 7).

Our patient presented with atypical clinical, polysomnographic, MRI, and CSF findings. The clinical course was dominated since the onset by fluctuating cognitive symptoms, improving after immunotherapy. The atypical clinical presentation might be caused by the fact that our patient has anti-IgLON5 antibodies exclusively of the IgG1 subclass, while to date all described cases have, to the best of our knowledge, presented with either isolated IgG4 or mixed IgG1 and IgG4 subtypes, with IgG4 predominance (1, 3). As IgG1 is able to bind complement, in contrast to IgG4 subtype (8), triggering of this activation route might be an explanation for the fierce inflammatory response seen in this patient. While we cannot completely exclude a viral encephalitis as a precipitating event, as there was no CSF analysis during the first presentation, this seems unlikely as the patient improved on treatment with steroids, which would aggravate an infectious cause. Also, the brain biopsy was not suggestive for an infectious pathology. Occurrence of NMDA-receptor encephalitis after herpes simplex encephalitis has been reported, leading to the hypothesis that the viral encephalitis was the triggering event for the development of the autoimmune encephalitis (9). Conversely, as the different subclasses of IgG can be produced in response to antigens dependently on the mechanism of sensitization, a possible preceding viral infection, not yet described in relation to IgLON5-associated encephalitis, could account for the production of IgG exclusively of class 1 in our patient (10). Typical sleep phenomena were not present in our patient though the sleep EEG was of the “undifferentiated non-REM-sleep” type. Although the presentation, clinical course, and MRI findings of this patient seem more compatible with GABA-A receptor encephalitis (11), these antibodies were undetectable, as were other known autoantibodies related to autoimmune encephalitis. While one might argue that this patient may have another unknown autoantibody implicated in the disease course, the fact that our patient has the same haplotype as in previously described cases as well as the presence of IgLON5 antibodies in serum and CSF suggests that the clinical spectrum of IgLON5-associated encephalitis is broader than what is known to date.

Our patient was treated with corticosteroids and with plasma exchange: after the latter treatment, her cognitive deficits improved dramatically and her anti-IgLON5 serum titer decreased from 1:10,000 to 1:320: this suggests that in our patient cognitive decline could be related to the titer of anti-IgLON5 antibodies and that the antibodies play a role in the pathogenesis. Moreover, the favorable treatment response to immunotherapy in this patient might also be related to the presence of IgG1 subclass antibodies, with effective removal of complement factors contributing to the treatment effect (12).

The early recognition of this autoimmune encephalitis and rapid treatment with corticosteroids and plasmapheresis may have resulted in the good outcome of this patient, compared to the non-response to immunotherapy in previously described cases. This supports the hypothesis that the antibodies are pathogenic and that neurodegeneration might be prevented by early treatment. Our patient presented with clear inflammatory changes on CSF and MRI, which might be another reason for her dramatical improvement after immunotherapy.

## Concluding Remarks

IgLON5-associated encephalitis is a relatively new autoimmune encephalopathy that can present with various neurological symptoms. This case report expands the clinical spectrum of this disease and supports the use of early immunotherapy.

## Informed Consent

The patient subject of this case report gave her written informed consent for the writing and publication of this case report.

## Ethics Statement

The ethics committee was not consulted. Written informed consent was obtained from the patient.

## Author Contributions

MM was involved in the patient case, collected necessary data, drafted and finalized the manuscript. RA was involved in the patient case, delivered necessary data, and critically revised the manuscript for intellectual content. IV was involved in the patient case and critically revised the manuscript for intellectual content. ML revised the brain biopsy, provided images of the specimen, and critically revised the manuscript for intellectual content. JH revised all brain MRIs, provided a selection of images, and critically revised the manuscript for intellectual content. BW was involved in the patient case, drafting of the manuscript and critically revision of different versions for intellectual content. All the authors approved the final version of this manuscript.

## Conflict of Interest Statement

The authors declared no potential conflicts of interest with respect to the research, authorship, and/or publication of this article. The authors received no financial support for the research, authorship, and/or publication of this article.
